# Deciphering the pathway-specific regulatory network for production of ten-membered enediyne Tiancimycins in *Streptomyces *sp. CB03234-S

**DOI:** 10.1186/s12934-022-01916-z

**Published:** 2022-09-10

**Authors:** Manxiang Zhu, Fan Zhang, Ting Gan, Jing Lin, Yanwen Duan, Xiangcheng Zhu

**Affiliations:** 1 Xiangya International Academy of Translational Medicine, Central South University, Tongzipo Road, #172, Yuelu District, Changsha, 410013 Hunan China; 2 Hunan Engineering Research Center of Combinatorial Biosynthesis and Natural Product Drug Discovery, Changsha, 410013 Hunan China; 3 National Engineering Research Center of Combinatorial Biosynthesis for Drug Discovery, Changsha, 410013 Hunan China

**Keywords:** Anthraquinone-fused enediynes, Tiancimycins, Orphan two-component regulatory system, Pathway-specific cascade regulatory network, EMSA, qRT-PCR

## Abstract

**Background:**

The anthraquinone-fused 10-membered enediynes (AFEs), represented by tiancimycins (TNMs), possess a unique structural feature and promising potentials as payloads of antitumor antibody–drug conjugates. Despite many efforts, the insufficient yields remain a practical challenge for development of AFEs. Recent studies have suggested a unified basic biosynthetic route for AFEs, those core genes involved in the formation of essential common AFE intermediates, together with multiple regulatory genes, are highly conserved among the reported biosynthetic gene clusters (BGCs) of AFEs. The extreme cytotoxicities of AFEs have compelled hosts to evolve strict regulations to control their productions, but the exact roles of related regulatory genes are still uncertain.

**Results:**

In this study, the genetic validations of five putative regulatory genes present in the BGC of TNMs revealed that only three (*tnmR1*, *tnmR3* and *tnmR7)* of them were involved in the regulation of TNMs biosynthesis. The bioinformatic analysis also revealed that they represented three major but distinct groups of regulatory genes conserved in all BGCs of AFEs. Further transcriptional analyses suggested that TnmR7 could promote the expressions of core enzymes TnmD/G and TnmN/O/P, while TnmR3 may act as a sensor kinase to work with TnmR1 and form a higher class unconventional orphan two-component regulatory system, which dynamically represses the expressions of TnmR7, core enzymes TnmD/G/J/K1/K2 and auxiliary proteins TnmT2/S2/T1/S1. Therefore, the biosynthesis of TNMs was stringently restricted by this cascade regulatory network at early stage to ensure the normal cell growth, and then partially released at the stationary phase for product accumulation.

**Conclusion:**

The pathway-specific cascade regulatory network consisting with TnmR3/R1 and TnmR7 was deciphered to orchestrate the production of TNMs. And it could be speculated as a common regulatory mechanism for productions of AFEs, which shall provide us new insights in future titer improvement of AFEs and potential dynamic regulatory applications in synthetic biology.

**Supplementary Information:**

The online version contains supplementary material available at 10.1186/s12934-022-01916-z.

## Background

**Fig. 1:**
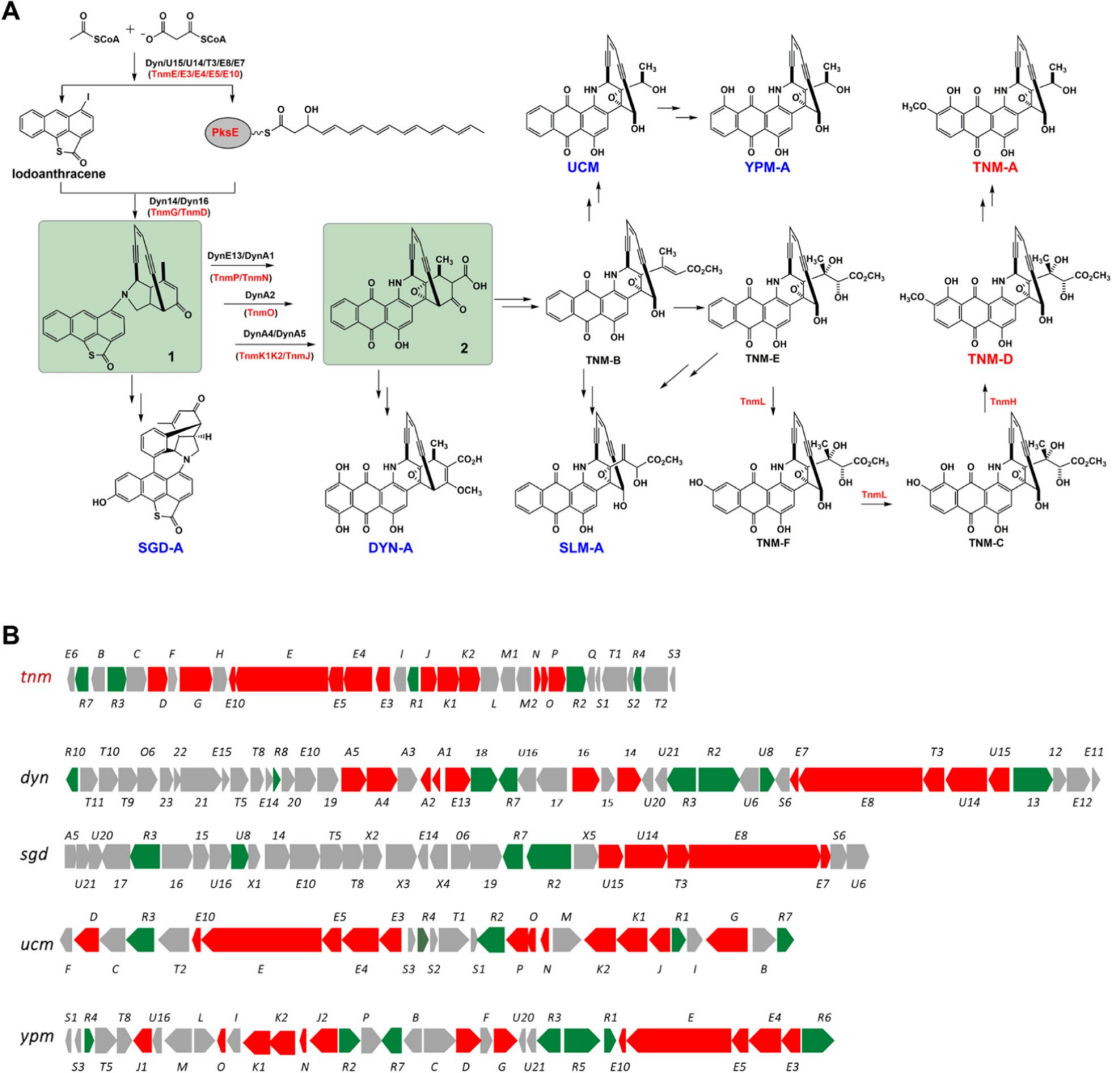
Summarized biosynthetic studies of AFEs: **A** The putative unified biosynthetic pathway with the formation of two essential AFE intermediates (highlighted by shadow) to generate different groups of AFEs, the involved core enzymes or their homologs were indicated; **B** The reported BGCs of AFEs with marked core genes (red) and putative regulatory genes (green)

Enediynes are a class of highly cytotoxic nature products with promising drug development potentials, especially as potent payloads for antitumor antibody–drug conjugates (ADCs) [1]. Among the existing enediynes, the anthraquinone-fused 10-membered enediynes (AFEs) possess a unique structure feature with a anthraquinone moiety and an enediyne core in one compact molecule [2], in which the anthraquinone moiety is presumed to not only conjugately stabilize the enediyne core, but also play an important role in the interaction with DNA and associated biological activities [3]. So far, there are six groups of known AFEs with an aromatized one, which include Yangpumicins (YPMs) [4, 5], Uncialamycin (UCM) [6], Sungeidines (SGDs) [7], Dynemicins (DYNs) [8], Sealutotomycins (SLMs) [9] and Tiancimycins (TNMs) [10], but only five of them have reported biosynthetic gene clusters (BGCs) (Fig. 1). Recent studies have revealed the prospects of AFEs in development of antitumor ADCs [1], but the insufficient yields of AFEs become a practical challenge to limit their prospective applications [11]. Despites many efforts [12, 13], the improvement of the titers of typical AFEs such as TNMs and YPMs remains too limited to satisfy practical requirements for industrialization. Since AFEs are causing DNA breakage and thus are lethal for their producers. Such potent inherent toxicity has forced the producers to evolve complex regulatory network to strictly control their productions.

Owing to the unique structural feature and the promising prospects in drug development, extensive studies have been addressed to the biosynthesis of AFEs, mainly on DYNs, SGDs and TNMs [11]. Although not fully elucidated, the solved major biosynthetic steps of DYNs [11] have revealed a unified principal pathway for AFEs biosynthesis (Fig. 1A). Briefly, the key precursor iodoathracene of anthraquinone moiety and the 10-membered enediyne core are both synthesized by the conserved enediyne polyketide synthase cassette (DynE7/E8/T3/U14/U15) [14, 15]. These two parts are enzymatically coupled (Dyn14/16) to form the pivotal AFE intermediate **1** ^15^, which then either shunts into a degenerative pathway to produce SGDs through a series of rearrangements, or undergoes further multi-step enzymatic reactions (DynE13/A1/A2/A4/A5) to generate a core AFE intermediate **2** [16]. All AFEs except SGDs are derived from **2** by various post-modification steps [1], including TNMs [17, 18]. It is noteworthy that iodine is essential for biosynthesis of AFEs because of the formation of key precursor iodoathracene [15]. Despite of different tailoring genes, the core genes involved in the biosynthesis of AFE scaffold, as well as multiple putative regulatory genes, are highly conserved among all AFE BGCs (Fig. 1B), whereas the nature of tailoring genes varies. Therefore, the productions of AFEs may share similar regulatory features.

In *Streptomycetes*, productions of secondary metabolites, especially antibiotics, are usually under the controls of various pleiotropic and pathway-specific regulators [19], in which the former ordinarily impact the whole metabolic network while the latter often directly control the target biosynthetic pathway. As the major type of pleiotropic regulator, two-component regulatory system consists of sensor kinase (SK) and response regulator (RR), which are usually adjacent together to sense and respond to various stresses [20], such as the well-known PhoR-PhoP system [21]. Two-component regulatory systems are important for signal transductions and generally participate in different global regulations, while a few of them could also modulate antibiotic productions like cephamycin C [22], salinomycin [23], oxytetracycline [24] and etc. On the other hand, the manipulation of pathway-specific regulator is a general strategy used for titer improvement of many important antibiotics such as platensimycin, a 9-membered enediyne C-1027 [25]. The progresses on biosynthetic and genomic studies have revealed that the regulation of antibiotic biosynthetic pathways was more complex than anticipated. For instance, the production of polyene antibiotic candicidin was shown to be controlled by a hierarchical regulatory network consisting of four regulators [26, 27]. The existence of multiple different regulatory genes in AFE BGCs (Fig. 1B) suggested the existence of regulatory cascades governing AFEs productions. Therefore, the exploration of cryptic AFE pathway regulatory networks will enable us to gain more insights in the regulation of AFEs biosynthesis and that is essential for their future titer improvement.

In previous work, we have obtained a ribosome engineering mutant *Streptomyces* sp.CB03234-S with the enhanced titers of TNMs (mainly TNM-A/D) [28], and revealed a global regulator WblA as the key factor repressing TNMs biosynthesis in CB03234-S [29]. On the basis of these preliminary results, TNMs biosynthesis was chosen as a model to investigate general pathway-specific regulatory mechanisms of AFEs biosynthesis. In this work, five putative regulatory genes found in the BGC of TNMs (*tnm*) were first genetically characterized to evaluate their potential effects on the cell growth and the production of TNMs in CB03234-S. Then a series of transcriptional analyses were carried out to decipher possible pathway-specific regulations in the biosynthesis of TNMs. Finally, an unconventional but stringent regulatory network for the production of TNMs was proposed. Our results unveiled a generic regulatory network for production of AFEs, which shall provide new insights in future titer improvement of AFEs and potential dynamic regulatory applications in synthetic biology.

## Results and discussion

### Genetic characterization of putative TNM pathway-specific regulators

**Fig. 2:**
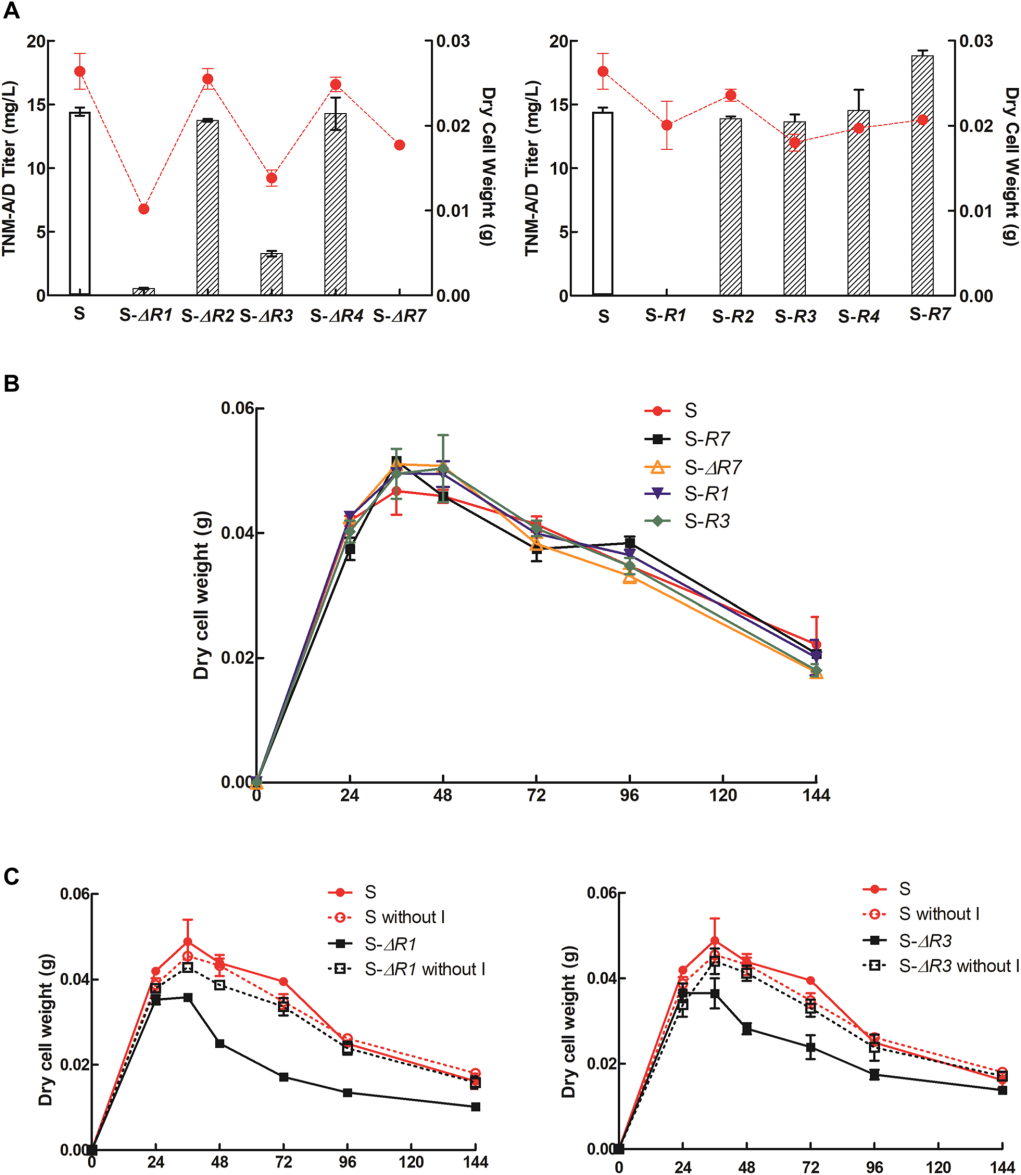
Genetic characterization of putative TNM pathway-specific regulators: **A** The comparisons of TNMs titer (bar chart) and corresponding biomass (dot with dash line) at the seventh day; **B** The comparison of growth curves from S, the overexpression mutants of *tnmR1* (S-*R1*), *tnmR3* (S-*R3*) and *tnmR7* (S-*R7*), as well as the knockout mutant of *tnmR7* (S-*ΔR7*); **C** The comparison of growth curves from S and the knockout mutant of *tnmR1* (S-*ΔR1*) or *tnmR3* (S-*ΔR3*) cultivated with or without iodine

The 5 putative *tnm* regulatory genes [11] were classified based on the amino acid sequence alignment and conserved domain search in NCBI (Additional file 1: Fig. S1). This analysis revealed that *tnmR1* encodes an HxIR family transcriptional regulator, *tnmR7* and *tnmR4* encode AraC family transcriptional regulators, *tnmR2* and *tnmR3* encoded AarF/ABC1/UbiB kinase family proteins. Each *tnm* regulatory gene was respectively knocked out or overexpressed in CB03234-S (Additional file 1: Fig. S2) to evaluate their possible influence on the production of TNMs. The fermentation results of derived mutants suggested that only TnmR1, TnmR3 and TnmR7 were involved with the production of TNMs (Fig. 2A). Among them, TnmR7 was a typical positive regulator since the production of TNMs was improved 1.3-fold by its overexpression but abolished by its deletion, whereas the roles of TnmR1 and TnmR3 were somewhat elusive. The deletion of TnmR3 evidently reduced the titer of TNMs for about fivefold, but its overexpression had no obvious effect, while either deletion or overexpression of TnmR1 could significantly decrease the production of TNMs.

Subsequent measurements of dry cell weight (DCW) indicated that the overexpression of any of the three regulators, or the deletion of TnmR7 had no apparent impact on the biomass of CB03434-S (Fig. 2B). In contrast, the deletion of either TnmR1 or TnmR3 notably impaired cell growth during exponential phase (24 h–48 h) and caused rapid cell death. Such deleterious effects could be totally relieved when the corresponding mutants were cultivated in absence of iodine (Fig. 2C). Therefore, the reduced titer of TNMs in TnmR1 or TnmR3 deletion mutant could be attributed to the substantial decrease of biomass. Because iodine is essential for the formation of key AFE precursor iodoathracene [15], the observed growth inhibition could be correlated with the intracellular accumulation of TNMs, which were extremely cytotoxic, prompting DNA breakage and thus inducing cell death. Hence, we speculated that TnmR1 and TnmR3 act together as a negative regulatory system to restrict the production of TNMs, so the deletion of either one would allow the biosynthesis of TNMs at the early stage of fermentation, resulting into premature cell death.

The five reported AFE BGCs all contain 4 to 8 putative pathway-specific regulatory genes belonging to different types and present at distinct locations in each BGC (Fig. 1B, Additional file 1: Table S3). The sequence similarity network (SSN) analysis at an E-value threshold of 1 × 10^−20^ grouped these 29 genes into 5 clusters (Additional file 1: Fig. S3). Among them, the 3 major clusters represented by *tnmR1*, *tnmR3* and *tnmR7* were conserved in all BGCs of AFEs. This bioinformatic analysis together with the outcome of our genetic studies suggested that the productions of AFEs may share a similar regulatory mechanism. So, the exploration of regulatory network in the production of TNMs is of a certain significance to acquire better understandings of biosynthesis of AFEs and guide their future industrial development.

### Determination of transcription units in *tnm* and subsequent qRT-PCR analyses of various regulatory gene mutants

**Fig. 3:**
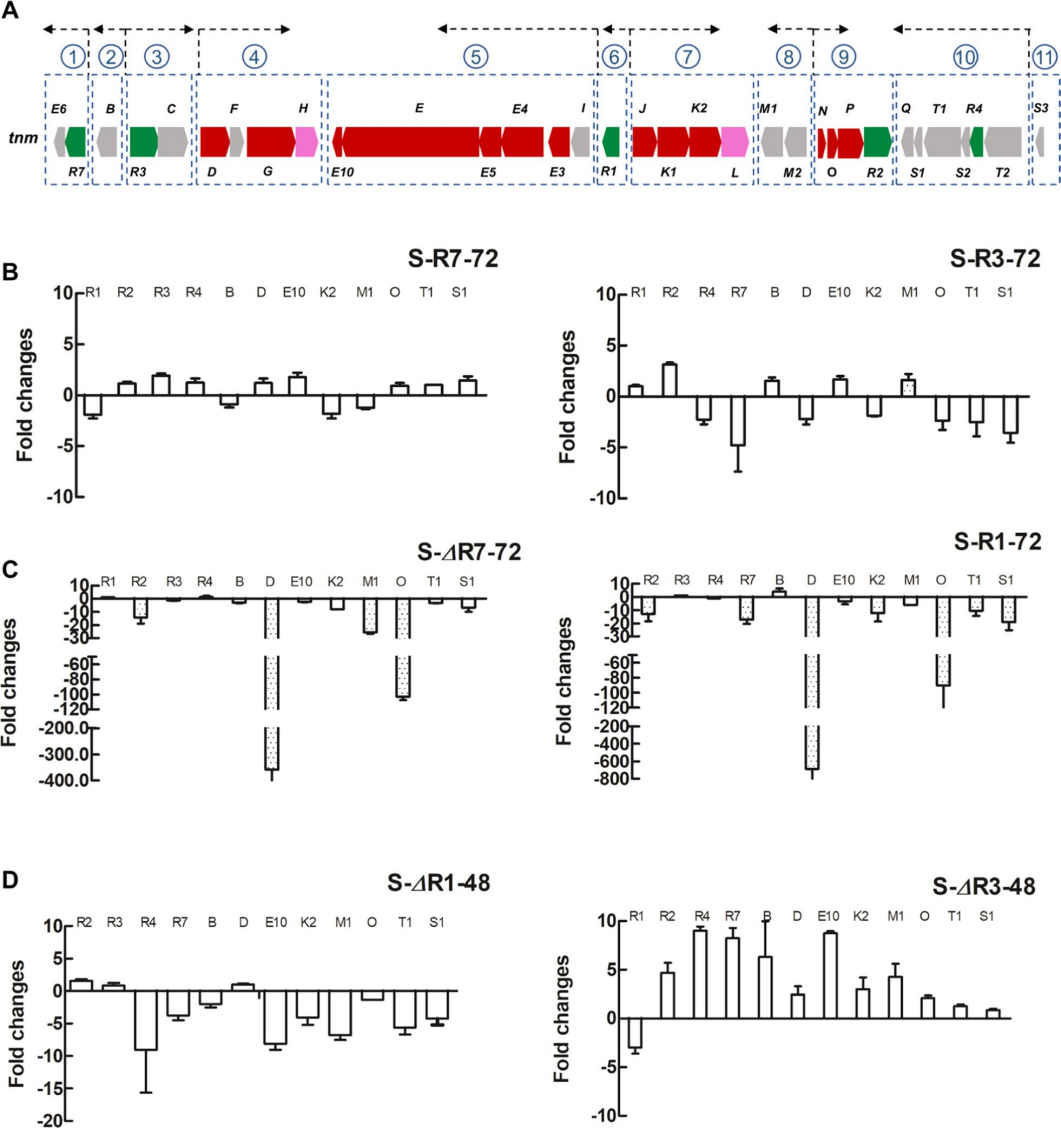
Exploration of potential transcriptional regulations on biosynthesis of TNMs: **A** The determination of possible transcription units in *tnm*; **B** The qRT-PCR results of S-*R7* and S-*R3* at 72 h; **C** The qRT-PCR results of S-*ΔR7* and S-*R1* at 72 h; **D** The qRT-PCR results of S-*ΔR1* and S-*ΔR3* at 48 h

Due to the diversity of gene arrangements, the possible transcription units in *tnm* were first determined by RT-PCR. Among all 25 intergenic regions found in *tnm*, those being between the "tail to tail" adjacent genes, or being less than 50 bp long were excluded, the remaining 13 intergenic regions contain promoters were subjected for RT-PCR analysis (Additional file 1: Table S4). Using cDNA originating from CB03234-S as a template, any intergenic region embodied in transcription unit would give a clear PCR product, whereas those failing to generate PCR fragments could constitute promoter areas delimiting different transcription units. Our RT-PCR results revealed 7 promoter regions delimiting 7 transcription units (Additional file 1: Fig. S4), plus 3 natural intervals between the "tail to tail" adjacent genes delimiting 4 transcription units. The TNM biosynthetic pathway could thus be divided into 11 possible transcription units (Fig. 3A). At least one gene was chosen from each transcription unit except for the last one. In total, 13 genes encoding biosynthetic core enzymes, tailoring enzymes, transport/resistance proteins and regulators, were selected for further qRT-PCR analyses in the different regulatory mutants.

The mRNA samples of *tnmR1* or *tnmR3* knockout mutant were collected ahead at 48 h because of their premature deaths, whereas the other mRNA samples, including those of reference strain CB03234-S were collected after 72 h cultivation. The expression level of target gene enhanced or decreased more than tenfold was considered as significant. As the results, no obvious expression changes on the majority of target genes were observed either in *tnmR2* and *tnmR4* related mutants (Additional file 1: Fig. S5), or the overexpression mutants of *tnmR7* and *tnmR3* (Fig. 3B). By contrast, the expressions of some target genes such as *tnmR2* and *tnmM1* were evidently repressed in S-*ΔR7,* especially *tnmD* and *tnmO* with over 350-fold and 100-fold reductions, respectively. Similar down-regulations but involved more genes were also found in S-*R1*, in which *tnmR7*, *tnmK2*, *tnmT1* and *tnmS1* should be directly controlled by TnmR1, while other genes might be hierarchically regulated through the mediation of TnmR7 (Fig. 3C). Most notably, the down-regulation of *tnmO* in both two mutants was in the same level, but the expression of *tnmD* was further decreased in S-*R1* to almost 700-fold, suggesting a potential superposed regulatory effect of TnmR1 on *tnmD*. On the other hand, the gene expression changes at 48H in S-*ΔR1* and S-*ΔR3* were almost opposite (Fig. 3D). Most of genes showed a up-regulated trend in S-*ΔR3*, which was reasonable due to the impairment of the negative regulation system; whereas the repressed trend of these genes in S-*ΔR1* could probably more relate to the premature death of cells, which was in accord with the lower biomass and titer of S-*ΔR1*.

Based on qRT-PCR results, we deduced that TnmR7 could promote the transcriptions of the fourth (*tnmDFGH*), eighth (*tnmM1M2*) and ninth (*tnmNOPR2*) units, while TnmR1 (probably with the assistance of TnmR3) could repress the transcriptions of the first (*tnmR7E6*), seventh (*tnmJK1K2L*) tenth (*tnmT2R4S2T1S1Q*) and possibly fourth (*tnmDFGH*) units. Referring to the proposed common pathway of AFEs, TnmD/G are responsible for the coupling of iodoathracene and enediyne core to form the AFE intermediate **1**, TnmN/O/P/J/K1K2 are involved with the transformation from **1** to the AFE intermediate **2**, which are two critical steps for the biosynthesis of TNMs (Fig. 1A). Therefore, TnmR1 and TnmR7 played important but opposite roles in the regulation of the formation of two crucial AFE intermediates, and thus impacted the production of TNMs. Besides, TnmR1 was on the higher level in this cascade regulatory network, it not only hierarchically controlled TnmR7, but also governed the expressions of core enzymes, as well as auxiliary proteins that are important for the resistance and transport of TNMs.

### Establishment of the possible regulatory network for the production of TNMs

To validate the potential bindings between seven promoter regions of transcription units and two regulators TnmR1 and TnmR7, the electrophoretic mobility shift assay (EMSA) [30] was subsequently carried out. Either *tnmR1* or *tnmR7* was first heterologously expressed with the fusion of typical His-tag at the C-terminal. The 28.7 kD TnmR1-His_6_ was successfully obtained and purified in the soluble form, but the 34.7 kD TnmR7-His_6_ was insoluble and could not be resolubilized (Additional file 1: Fig. S6). The hydrophilicity analysis of TnmR7 revealed that its N-terminal part bears very hydrophobic region, so different truncated forms of *tnmR7* retaining the intact HTH domain with the entire N-terminal or without the hydrophobic region (Additional file 1: Fig. S7A) were expressed. Despite numerous trials under various conditions including different temperatures and the addition of different amount of IPTG, we could not obtain soluble proteins (Additional file 1: Fig. S7A). Meanwhile, different molecular chaperones, such as PGR07, PTF16 and PGT12, or the pGEX-2T fusion system containing glutathione-S-transferase, were individually adopted, but all failed to make the soluble expression of TnmR7 (Additional file 1: Fig. S7B). Therefore, only TnmR1-His_6_ was subjected to the EMSA by binding with biotin-labeled promoter regions**.**

**Fig. 4:**
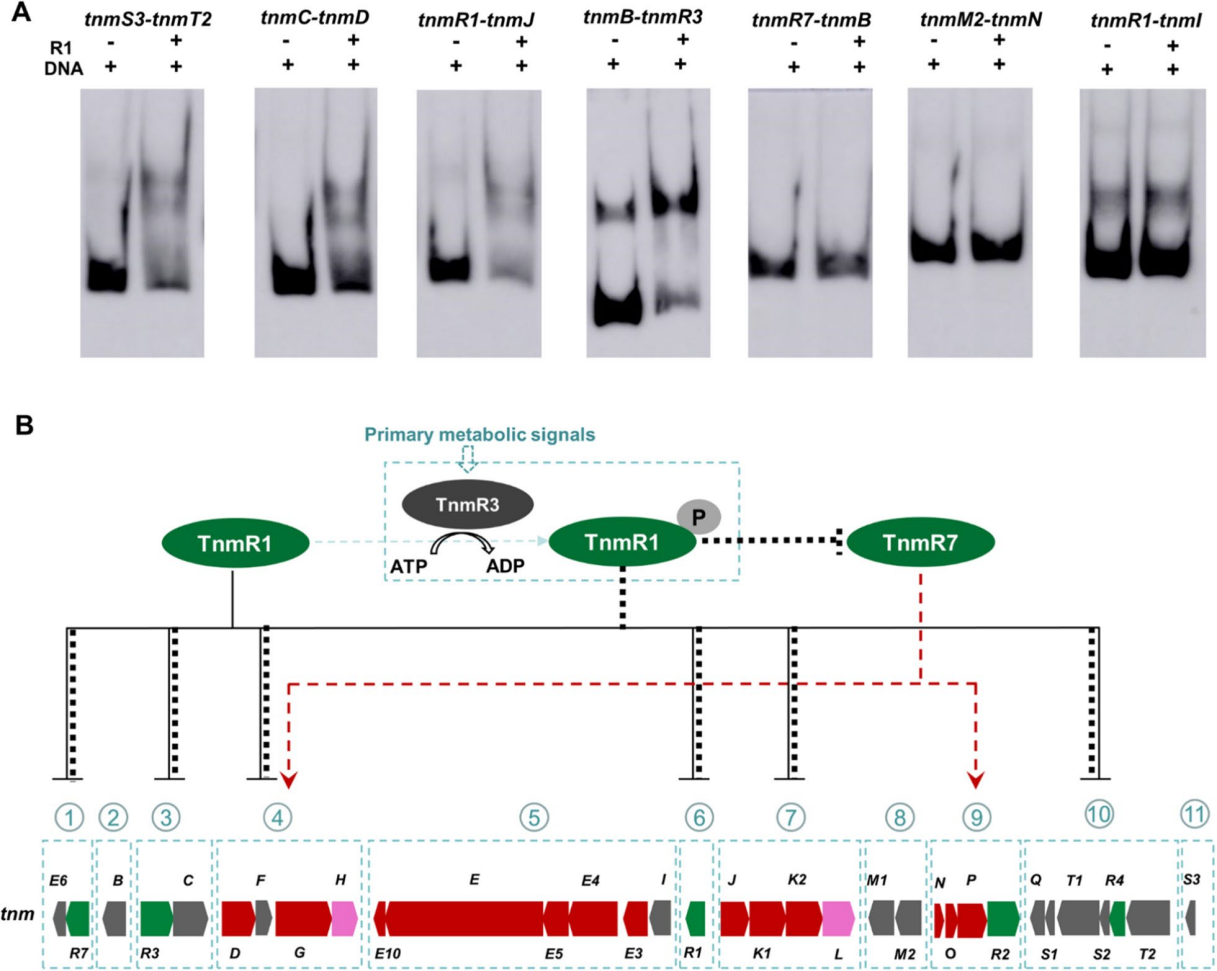
Establishment of a possible regulatory network for the production of TNMs: **A** The EMSA results of 7 biotin-labeled promoter regions with or without the presence of TnmR1; **B** The proposed delicate regulatory network to control the biosynthesis of TNMs (the black solid line means the results of TnmR1 were validated by both qRT-PCR and EMSA, the red dash line means the results of TnmR7 were deduced from qRT-PCR, the black bold dash line indicates the enhanced regulations of phosphorylated TnmR1, the blue dash box means the result was speculated from the genetical characterization)

Among seven target promoter regions, TnmR1 showed a clear but only moderate interaction with *tnmB-tnmR3* (between unit 2 and 3), *tnmC-tnmD* (between unit 3 and 4), *tnmR1-tnmJ* (between unit 6 and 7) and *tnmT2-tnmS3* (between unit 10 and 11), respectively. In contrast, TnmR1 had no interaction with tnmI-tnmR1 (between unit 5 and 6) and tnmM2-tnmN (between unit 8 and 9) (Fig. 4A). These EMSAs results were basically consistent with the qRT-PCR data obtained with the TnmR1 mutant. It was noteworthy that single TnmR1 seemed to only partially bind with target promoter regions. Although the real function of TnmR3 was not validated, its deletion phenotype was very similar to that of TnmR1, which suggested that TnmR3 may act as an orphan sensor kinase to enhance the regulatory effect of TnmR1, and they together formed a special two-component regulatory system to strictly control the biosynthesis of TNMs. Although two-component regulatory systems are abundant in streptomycetes and could affect antibiotic production through cascade regulations [31], they have seldom been encoded by genes situating in BGCs and acted as pathway-specific regulators. It had been reported that *lipReg1/2* in the BGC of acyclic polyene α-lipomycin were encoding a putative two-component regulatory system, but LipReg1 and LipReg2 just indirectly influenced the production of α-lipomycin through a pathway-specific regulator LipReg4 [32]. Therefore, TnmR1 and TnmR3, as well as their homologs from other AFEs, represented a rare type of unconventional orphan two-component regulatory system to directly control the biosynthesis of AFEs like TNMs.

Based on the obtained overall results, a cascade regulatory network, which consisted with a higher class negative two-component regulatory system TnmR3/R1 and a positive regulator TnmR7, was proposed to orchestrate the production of TNMs (Fig. 4B). During the growth stage, TnmR3 could sense primary metabolic signals to phosphorylate TnmR1, which then tightly bind with target promoter regions to repress the transcriptions of core enzymes TnmJ/K1/K2, the resistance & transport system TnmT2/S2/T1/S1 and the negative control system itself. Besides, the phosphorylated TnmR1 could not only compete with TnmR7 at the same promoter region to down-regulate the expressions of core enzymes TnmD/G, but also disturb the transcription of TnmR7 to repress the expressions of another group of core enzymes TnmN/O/P. Since these core enzymes are essential for the formation of key AFE intermediates **1** and **2**, the stringent regulation on their transcriptions by TnmR3/R1 could inhibit the production of lethal TNMs and therefore ensure the normal growth of host cells. When the primary metabolism is declined at the stationary stage of fermentation [33], the changes of primary metabolic signals could make TnmR3 inert, and the initial TnmR1 with the much weaker binding ability is unable to persist the strict regulation. As a result, the transcriptions of those core enzymes as well as the positive regulator TnmR7 are partially released and thus enabled the production of TNMs.

## Conclusion

In conclusion, our work revealed that the production of highly toxic TNMs, a group of potential antitumor ADC payloads, was stringently controlled by the pathway-specific cascade regulatory network, which consists with a higher class unconventional orphan two-component regulatory system TnmR3/R1 and a positive regulator TnmR7. As the principal part of regulatory network, TnmR3/R1 mainly repressed the transcriptions of different proteins related to the biosynthesis of TNMs, including TnmR7 and core enzymes involved with the formation of essential common AFE intermediates, as well as auxiliary proteins important for the resistance and transport of TNMs. Besides that, TnmR3/R1 was proposed to dynamically regulate the expressions of target genes by sensing the change of primary metabolic signals. Through this delicate regulatory network, the host could tactfully avoid the early production of TNMs and accompanying lethal effects to ensure the normal growth of cells.

Since the biosynthesis of AFEs are suggested to share a unified principal pathway, and the homologues of *tnmR1*, *tnmR3* and *tnmR7* are highly conserved in all BGCs of AFEs, the deciphered pathway-specific regulatory network of TNMs could be speculated as a common regulatory mechanism for the existed AFEs, which shall provide us new insights to understand and manipulate the biosynthesis of AFEs, and contribute to their future synthetic biology development.

## Materials and methods

### Bacterial strains and culture conditions

*S.* sp. CB03234-S was obtained from the previous work [28] and reserved in our lab. The complete strains and plasmids used or constructed in this study were listed in Additional file 1: Table S1. All designed primers were listed in Additional file 1: Table S2. As reported before [28, 34], Gauze’s (G1) solid medium was applied for sporulation; mannitol soya flour (MS) solid medium was applied for intergeneric conjugation; the tryptic soy broth (TSB) seed medium and optimal production (OP) medium were applied for liquid fermentation, with the supply of 1.5% (w/w) Dianion HP20 resins in OP medium. Various *Escherichia coli* strains were cultured in Luria − Bertani (LB) medium for cloning, conjugation and protein expression, respectively. Different antibiotics (50 mg/L apramycin, or 60 mg/L streptomycin, or 20 mg/L thiostrepton, or 30 mg/L kanamycin) were correspondingly added when necessary.

### Construction of mutants related to five regulatory genes from *tnm* BGC in CB03234-S

Each target gene was amplified from the genomic DNA of CB03234-S by using the high-fidelity Golden PCR Mix TSE101 (Tsingke Biotech. Co., Changsha, China), and cloned into linearized pSET152 via the Trelief SoSoo seamless cloning kit (Tsingke) to construct the overexpression plasmid. To obtain the gene knockout plasmid, the amplified upstream and downstream regions (each 2.0 kb) of target gene, as well as the 919 bp thiostrepton resistance gene *tsr* with a constitutive promoter ermE*, were joined together with linearized pOJ260 through seamless cloning kit. The corresponding plasmid was introduced into CB03234-S by conjugation transfer. The resulting thiostrepton-resistant or apramycin-resistant exconjugants were verified by PCR amplification and sequencing to finally obtain expected target mutants.

### Fermentation, HPLC analysis and biomass determination in target mutants

The spore suspension of each mutant or CB03234-S was inoculated into 50 mL seed medium and grown at 220 rpm, 30 °C for 36H. Then, 10% (v/v) inocula were transferred into 50 mL OP medium and cultivated at the same conditions for 7 days. The resulted mycelia and resins were collected and treated with 50 mL methanol, and the extracts were subjected to HPLC analysis as previously described [29]. For biomass determination, the target strain was cultivated in OP medium without CaCO_3_ and resins, and sampled 5 mL at 24 h, 36 h, 48 h, 72 h, 96 h and 144 h, respectively. After centrifugation at 4000 rpm for 10 min, the obtained cell pellets were dried to a constant weight at 90 °C to determine the biomass. All experiments were carried out at least in triplicate, the data were statistically analyzed using GraphPad Primer 5.0 and presented as mean ± SD.

### Transcriptional analyses of CB03234-S and derived mutants

CB03234-S or the derived mutant was cultivated in OP medium for 72 h or 48 h to collect cells by centrifugation at 4000 rpm for 10 min. According to the manufactural instructions, the total RNA of target strain was isolated by using RNAiso Plus (Takara, Dalian, China), and reverse transcribed into cDNA by using Goldenstar RT6 cDNA Synthesis Mix TSK314S (Tsingke). Either genomic DNA or cDNA of CB03234-S was applied as the template for PCR amplification to determine the transcription unit. Quantitative real-time PCR (qRT-PCR) [35] was carried out by using SYBR Green I Fast qPCR Mix TSE202 (Tsingke) and designed primers in Roche LightCycler96 thermal cycler. The relative quantities of target cDNA were normalized by the conservative *hrdB*, which encodes the major sigma factor in *Streptomyces*.

### Protein expression and electrophoresis mobility shift assay (EMSA)

The 744 bp *tnmR1* was amplified and cloned into pET-28a through *Nde*I/*Hind*III sites, then pET28a-R1 was introduced into *E. coli* BL21 (DE3) and expressed at 30 °C by induction of 0.1 mM IPTG for 15 H. The resulting soluble fraction was purified by using High Affinity Ni–NTA Resins (GenScript Inc., Nanjing, China) to obtain the pure soluble TnmR1-His_6_, which was then confirmed by SDS-PAGE analysis and quantified by BCA Protein Assay Kit (Sangon biotech., Shanghai, China). Similarly, the 915 bp *tnmR7* was first cloned into pET-28a and tried different induction conditions (IPTG concentration and temperature) in BL21(DE3) for protein expression. The inclusion body of TnmR7-His_6_ was renatured following a common protocol[36]. Meanwhile, *tnmR7* was cloned into pGEX-2 T fusion system containing glutathione-S-transferase, or co-expressed with different molecular chaperone expression plasmids including pGR07, pTF16 and pGT12 to try to get soluble TnmR7. And three truncated *tnmR7* fragments (402 bp, 525 bp and 717 bp) were respectively amplified and cloned into pET-28a to attempt soluble expression. For EMSAs, seven target promoter regions were ligated into pUC19 and amplified using the universal primer M13-R/F with the biotin label to obtain the biotin-labeled probes. By applying the commercial EMSAs kit (Viagene Biotech. Inc., Changzhou, China), 600 ng TnmR1-His, 5 ng target probe, 10X binding buffer and Poly (dI-dC) were incubated together at room temperature for 20 min, and electrophoresed in a 5.5% gel. The resulted gel was transferred to the nylon membrane, and immobilized by UV cross-linkage. Labeled DNA was detected with streptavidin–horseradish peroxidase (HRP) and ECL, then exposed and photographed [37, 38].

## Supplementary Information

**Additional file 1: Figure.** Including BLAST alignment of five putative Tnm regulators, SNN analysis of putative pathway-specific regulatory genes from AFE BGCs, deletion and verification of putative *tnm* regulatory genes, RT-PCR analyses for determination of transcription unit in *tnm*, qRT-PCR analyses of *tnmR2* and *tnmR4* related mutants, SDS-PAGE analyses for heterologous expressions of TnmR1 and TnmR7; and tables listing the homolog alignment of putative regulators in AFEs, putative promoter regions of *tnm*, the strains, plasmids, and primers used in this manuscript, are all provided in the supporting information.

## Data Availability

All data generated or analyzed during this study are included in this published article and its Additional files Fig 1, Fig. 2, Fig. 3, Fig. 4 and Additional Information.

## Abbreviations

AFEs: Anthraquinone-fused 10-membered enediynes
TNMs: Tiancimycins
ADCs: Antibody–drug conjugates
YPMs: Yangpumicins
UCM: Uncialamycin
SGDs: Sungeidines
DYNs: Dynemicins
SLMs: Sealutotomycins
BGCs: Biosynthetic gene clusters
SK: Sensor kinase
RR: Response regulator
DCW: Dry cell weight
SSN: Sequence similarity network
EMSA: Electrophoretic mobility shift assay
G1: Gauze’s solid medium
MS: Mannitol soya flour solid medium
TSB: Tryptic soy broth seed medium
OP: Optimal production medium
