# Supplementary material for: Deciphering the pathway-specific regulatory network for production of ten-membered enediyne Tiancimycins in Streptomyces sp. CB03234-S
Source: Microb Cell Fact. 2022 Sep 10;21:188. doi: 10.1186/s12934-022-01916-z (PMC9464397; doi:10.1186/s12934-022-01916-z)
Supplement: Supplementary file 1 — Additional file 1: Figure. Including BLAST alignment of five putative Tnm regulators, SNN analysis of putative pathway-specific regulatory genes from AFE BGCs, deletion and verification of putative tnm regulatory genes, RT-PCR analyses for determination of transcription unit in tnm, qRT-PCR analyses of tnmR2 and tnmR4 related mutants, SDS-PAGE analyses for heterologous expressions of TnmR1 and TnmR7; and tables listing the homolog alignment of putative regulators in AFEs, putative promoter regions of tnm, the strains, plasmids, and primers used in this manuscript, are all provided in the supporting information. [file 12934_2022_1916_MOESM1_ESM.pdf]

Deciphering the pathway-specific regulatory network for production of ten-membered enediyne Tiancimycins in *Streptomyces sp* CB03234-S

Manxiang Zhu [1], Fan Zhang [1], Ting Gan[1]， Jing Lin [1], Yanwen Duan[* 1, 2, 3] and Xiangcheng Zhu[* 1, 2, 3]

Supplementary Data

[1]Xiangya International Academy of Translational Medicine, Central South University,

[2]Hunan Engineering Research Center of Combinatorial Biosynthesis and Natural Product Drug Discovery, and

[3]National Engineering Research Center of Combinatorial Biosynthesis for Drug Discovery, Changsha, Hunan, 410013, China;

[*]To whom correspondents should be addressed: Yanwen Duan, Email: ywduan66@sina.com or Prof. Xiangcheng Zhu, Email ： seanzhu1996@aliyun.com.

Table S1. Strains and plasmids used in this study

Strain/plasmid	Descriptions	Reference or source
**1. Mutant construction**		
E. coli. DH5α	General cloning	Commercial source
E. coli. S17-1	Intergeneric conjugal transfer	Commercial source
S. sp CB03234	Original producing strain of TNMs	[1]
S. sp CB03234-S	High producing strain of TNMs, Streptomycin^R	[2]
S-△*R1*	CB03234-S with the deletion of *tnmR1*, Thiostrepton^R	this study
S-△*R2*	CB03234-S with the deletion of *tnmR2*, Thiostrepton^R	this study
S-△*R3*	CB03234-S with the deletion of *tnmR3*, Thiostrepton^R	this study
S-△*R4*	CB03234-S with the deletion of *tnmR4*, Thiostrepton^R	this study
S-△*R7*	CB03234-S with the deletion of *tnmR7*, Thiostrepton^R	this study
S-*R1*	CB03234-S carrying pSET152- *tnmR1*, Apramycin^R	this study
S-*R2*	CB03234-S carrying pSET152- *tnmR2*, Apramycin^R	this study
S-*R3*	CB03234-S carrying pSET152- *tnmR3*, Apramycin^R	this study
S-*R4*	CB03234-S carrying pSET152- *tnmR4*, Apramycin^R	this study
S-*R7*	CB03234-S carrying pSET152- *tnmR7*, Apramycin^R	this study
pOJ260	Conjugal shuttle vector, Apramycin^R	Commercial source
pOJ260-△*R1*	pOJ260 harboring the up and downstream 2.0 kb regions of *tnmR1* integrated with *tsr*	this study
pOJ260-△*R2*	pOJ260 harboring the up and downstream 2.0 kb regions of *tnmR1* integrated with *tsr*	this study
pOJ260-△*R3*	pOJ260 harboring the up and downstream 2.0 kb regions of *tnmR1* integrated with *tsr*	this study
pOJ260-△*R4*	pOJ260 harboring the up and downstream 2.0 kb regions of *tnmR1* integrated with *tsr*	this study
pOJ260-△*R7*	pOJ260 harboring the up and downstream 2.0 kb regions of *tnmR1* integrated with *tsr*	this study
pSET152	Integrative conjugal shuttle vector, Apramycin^R	Commercial source
pSET152-*R1*	pSET152 harboring *tnmR1*	this study
pSET152-*R2*	pSET152 harboring *tnmR2*	this study
pSET152-*R3*	pSET152 harboring *tnmR3*	this study
pSET152-*R4*	pSET152 harboring *tnmR4*	this study
pSET152-*R7*	pSET152 harboring *tnmR7*	this study

Table S1 (continue). Strains and plasmids used in this study

Strain/plasmid	Descriptions	Reference or source
**2. Protein expression**		
E. coli. BL21(DE3)	Protein expression	Commercial source
pET-28a (+)	Expression vector with His-tag, Kanamycin^R	Commercial source
pET28a-*R1*	pET28a harboring *tnmR1*	this study
pET28a-*R7*	pET28a harboring *tnmR7*	this study
pET28a-*R7*-717	pET28a harboring 717 bp *tnmR7* fragment	this study
pET28a-*R7*-525	pET28a harboring 525 bp *tnmR7* fragment	this study
pET28a-*R7*-402	pET28a harboring 402 bp *tnmR7* fragment	this study
pGR07	molecular chaperone expression plasmid	commercial
pTF16	molecular chaperone expression plasmid	commercial
pTF12	molecular chaperone expression plasmid	commercial
pGEX-2T	Expression vector with GST fusion protein, Ampicillin^R	commercial
pGEX-2T-*R7*	pGEX-2T harboring *tnmR7*	this study

Table S2. Designed primers used in this study

Primer	Nucleotide sequence (5'-3')
**1. For mutant construction and verification**	
tnmR1-F	gcacaatcgtgccggttggtaggatccCGTTGGCCGAGATTGCTAC
tnmR1-R	cttgggctgcaggtcgactctagaCGTTGGCCGAGATTGCTAC
tnmR2-F	gcacaatcgtgccggttggtaggatccATGAACCGAGTACCGGTGC
tnmR2-R	cttgggctgcaggtcgactctagaCTACGAGCAGAACGGCTGAT
tnmR3-F	gcacaatcgtgccggttggtaggatccCTCATCGGTAAGTGTTTCCCAAGTC
tnmR3-R	cttgggctgcaggtcgactctagaATCGCCGCTCACCGGTTCA
tnmR4-F	acaatcgtgccggttggtaggatccATGCCAGGCCAAGAACCCG
tnmR4-R	cttgggctgcaggtcgactctagaATCATGAATGTCCTTCCGGGGTCGAT
tnmR7-F	acaatcgtgccggttggtaggatccATGCCCCCGTACCCGC
tnmR7-R	cttgggctgcaggtcgactctagaTCAGCCGCTGCCGGAGG
tnmR1-up-F	gcggccgcggatcctctagaCGTAGTCCAGGAACATCCGC
tnmR1-up-R	ccgttcgaatgtgaacaGCTCACCGATCACGTTCAG
tnmR1-down-F	gccaaccgataaAGTTCATGGGCCTGCTCG
tnmR1-down-R	acgacggccagtgccaagcttCTCCAGGAACCACAGGCTG
tnmR1-*tsr*-F	ggtgagcTGTTCACATTCGAACGGTCTCTG
tnmR1-*tsr*-R	ggcccatgaactTTATCGGTTGGCCGCGAG
tnmR1-YZ-F	GTCGAGCAGGCCCATGAAC
tnmR1-YZ-R	GGAGCTACGGCCAGTTCTG
tnmR2-up-F	gcggccgcggatcctctagaGACAATTCCATGACCAGCAGC
tnmR2-up-R	gttcgaatgtgaacaATCTGGGCGAACTTCACGAAC
tnmR2-down-F	gccaaccgataaAAGCTGATCGGGGAGTTCTTC
tnmR2-down-R	acgacggccagtgccaagcttTGTACCTCCAGCAGGTGCAC
tnmR2-*tsr*-F	gttcgcccagatTGTTCACATTCGAACGGTCTCTG
tnmR2-*tsr*-R	cccgatcagcttTTATCGGTTGGCCGCGAGA
tnmR2-YZ-F	GGAAGCGGTGGTGACCCTG
tnmR2-YZ-R	ATGCTCGCCAGCATGATCTC
tnmR3-up-F	acgacggccagtgccaagcttGTACGCCATACGGGTAGAGG
tnmR3-up-R	tgtgaacaGTCGCTGAGGAGCCGTAC
tnmR3-down-F	ggccaaccgataaCTGGAAGGGATGATCAACGACTTC
tnmR3-down-R	gcggccgcggatcctctagaGTCCGCGTACTCGAAGAGC
tnmR3-*tsr*-F	ggctcctcagcgacTGTTCACATTCGAACGGTCTCTG
tnmR3-*tsr*-R	tcccttccagTTATCGGTTGGCCGCGAGA
tnmR3-YZ-F	TCGGCTCGCCGTGATCAC
tnmR3-YZ-R	GAAGTCGTTGATCATCCCTTCC
tnmR4-up-F	gcggccgcggatcctctagaGTAAACGCATAGCGACCCAC
tnmR4-up-R	cgaatgtgaacaGGTTCTTGGCCTGGCATGA
tnmR4-down-F	caaccgataaGACCCCGGAAGGACATTCAT
tnmR4-down-R	acgacggccagtgccaagcttGAGACCACCTTGGACAGGTC
tnmR4-*tsr*-F	aggccaagaaccTGTTCACATTCGAACGGTCTCTG

Table S2 (continue). Designed primers used in this study

Primer	Nucleotide sequence (5'-3')
tnmR4-tsr-R	ccttccggggtcTTATCGGTTGGCCGCGAGA
tnmR4-YZ-F	CTCATGCCAGGCCAAGAAC
tnmR4-YZ-R	GTGCGCTCATGAATGTCCTTC
tnmR7-up-F	gcggccgcggatcctctagaTTGACGAACGTAGGCCCGA
tnmR7-up-R	gttcgaatgtgaacaTGGAACGGGCTGAAGAGGC
tnmR7-down-F	gccaaccgataaGTCTGGTTCGACCGTGACAC
tnmR7-down-R	acgacggccagtgccaagcttAGTCACAAAGGAGGCTGGAG
tnmR7-tsr-F	gcccgttccaTGTTCACATTCGAACGGTCTCTG
tnmR7-tsr-R	ggtcgaaccagacTTATCGGTTGGCCGCGAGA
tnmR7-YZ-F	CAGTGCCTCTCCGACCAC
**2. For transcriptional analyses**	
R7-B-F	TACGGCGCGTTCGACCGAG
R7-B-R	TCGTTCGTGCACCACGGAT
B-R3-F	GTGGTGGTCCGACATCTGTC
B-R3-R	CTCGCAGCGAGTCGAACAG
C-D-F	GGAGTGGTCGTTGTGGAGC
C-D-R	ACTTGTTGCACAAGTCGATAGCC
F-G-F	TACCTGCCCAAGGAGCAGTTC
F-G-R	ACGGTGACGGTGCTGACCA
E3-E4-F	AACCTGCTCGCGAGTTCT
E3-E4-R	CTGCTCTGGAGAGTGCTGATC
I-E3-F	GGAGGCCGGAACGTAAATGC
I-E3-R	CCTCAACGAGATACTGCTGCG
R1-I-F	TGAGTCGGTACTGCCGGC
R1-I-R	GGAACAGCCCGAGCTGGT
R1-J-F	TGAGTCGGTACTGCCGGC
R1-J-R	CATGTCGTCCAGGAAACGG
M2-N-F	TGGTTCTCGGAACGTTCGGA
M2-N-R	GCCAGATCCTCCGGCATAC
P-R2-F	CTTCGAACCGGCCTGTTG
P-R2-R	ATCTGGGCGAACTTCACGAAC
T1-S1-F	ATCGATCGTACCGATGTGCG
T1-S1-R	CTTCATGGACGGCTTCCACAC
T2-R4-F	GCGGTCTATCAGGTCCTTGG
T2-R4-R	TGATCCTCACCCTGTCCGTC
S3-T2-F	CCATCCGGTAGCCCATGTG
S3-T2-R	CAGGCCATGTTCAGTGACCC
tnmB-RT-R	AACGACAGCCGGGTTCTG
tnmB-RT-F	CGTCAGCGACTGGCAGCT

Table S2 (continue). Designed primers used in this study

**Primer**	**Nucleotide sequence (5'-3')**
tnmD-RT-R	ACAACCGCAGCAACGACG
tnmD-RT-F	GGTCGTTGCGGATGGCCT
tnmK2-RT-R	TGATCGTGGTCGGCATGCTCAA
tnmK2-RT-F	GCGCTCGTACAGGTCGACATTACG
tnmE10-RT-R	GAACGGGCTCGGGTTCCATG
tnmE10-RT-F	CATGGAGTACCTGCGCGAGT
tnmM1-RT-R	TAGCCCATGGAGTACGGGGTG
tnmM1-RT-F	GCTGCCCGAACTGCCCTA
tnmO-RT-R	AAAGGCCGCGTATGAGGAGACG
tnmO-RT-F	GCACACCGACGTCAAAACCTCC
tnmS1-RT-R	ACGGCTGGAGGAGCAGGAA
tnmS1-RT-F	CAAGGCCACCCACGAGGAA
tnmT1-RT-R	GATGATGCCCCAGACCAGCG
tnmT1-RT-F	GGCGCTGGGTGTTCTTCATCAA
tnmR2-RT-R	ATCGCCTGCGTATACCGGG
tnmR2-RT-F	CCAGGATCGCGTTGCTCACGTA
tnmH-RT-R	ACCTGCTGCGGCTCAAGG
tnmH-RT-F	CCGACCGTTGGGCCAGTT
tnmR7-RT-R	CGGTGCACACATCGGTGAC
tnmR7-RT-F	CCAGTGCCTCTCCGACCAC
tnmR3-RT-R	CGACCTGACCGCGTTCAAG
tnmR3-RT-F	GTCGTTGATCATCCCTTCCAGG
tnmR1-RT-R	CGATGCGCACGAAAGTGTC
tnmR1-RT-F	CCGTGCACGGTGTCGATGA
tnmR4-RT-R	CCGACGATCCCGGAGAAC
tnmR4-RT-F	CTTCCTGCGCTCCTTCCAG
hrdB-F	GCACATCCCGTACGCTCCCG
hrdB-R	CTCGAAGGCCCGACGCACGT
**3. For protein expression**	
pET28a-*R7*-R	tgcggccgcaagcttTCAGCCGCTGCCGGAGGCG
pET28a-*R7*-F	gcggcagccatatgATGCCCCCGTACCCGCCC
pET28a-*R1*-R	ccaagcttTCAGACGCTTCCGGAACCG (*Hind*III site)
pET28a-*R1*-F	cccatatgATGACGGGTAAGCGGAGCTACG (*Nde*I site)
pGEX-2T-*R7*-F	CTGGTTCCGCGTGGATCCATGCCCCCGTACCCGCCCGC
PGEX-2T-*R7*-R	GTCAGTCACGATGAATTCTCAGCCGCTGCCGGAGGCGGC
pET28a-*R7*-402-R	gtgcggccgcaagcttGGCGATGTCACCGATGCGGCG
pET28a-*R7*-402-F	CGCgcggcagccatatgATGCCCCCGTACCCGCCC
pET28a-*R7*-525-R	gtgcggccgcaagcttCAGCCCGCGGGCGTCGCG
pET28a-*R7*-525-F	cgcgcggcagccatatgATGCCCCCGTACCCGCCCGC

Table S2 (continue). Designed primers used in this study

Primer	Nucleotide sequence (5'-3')
pET28a-*R7*-717-R	gtgcggccgcaagcttGACCAGGCGGCGTTCGCCCGC
pET28a-*R7*-717-F	cgcgcggcagccatatgATGCCCCCGTACCCGCCCGC
R7-E-F	ccaagcttTCGTCGGCCGGTGATGC (*Hind*III site)
R7-E-R	cggaattcCTCGGCGGCACCGACTGAC (*EcoR*I site)
R3-E-F	ccaagcttGTGGTGGTCCGACATCTGTCCT (*Hind*III site)
R3-E-R	cggaattcGGCGCAGAGAAGTGGTCCAC (*EcoR*I site)
D-E-F	ccaagcttTGCGGACCTGACGCCTTGC (*Hind*III site)
D-E-R	cggaattcGCCATGTTCTGCGCTTCCTT (*EcoR*I site)
I-E-F	ccaagcttGATTCGGTCATGGTGCGAGTCC (*Hind*III site)
I-E-R	cggaattcGGAAGCGTCTGACGGTCCATCA (*EcoR*I site)
R1-E-F	ccaagcttTAGCTCCGCTTACCCGTCATGAC (*Hind*III site)
R1-E-R	cggaattcCACGGGTCTGAGCCTCCATCA (*EcoR*I site)
N-E-F	ccaagcttTTCTCGGAACGTTCGGACCCG (*Hind*III site)
N-E-R	cggaattcCCGCCAGATCCTCCGGCATA (*EcoR*I site)
T2-E-R	ccaagcttGTGCACATCGTCGTCCATGC (*Hind*III site)
T2-E-F	cggaattcGTCGGCCGGTTGACCTCT (*EcoR*I site)

Lowercase letters were the overlapping regions designed for seamless cloning.

Underlined lowercase letters were introduced restriction enzyme digestion sites with 2 bp protective nucleotides.

Table S3. Assumed pathway-specific regulators in AEFs BGCs

predicted functions	*dyn* genes	*tnm* Genes	*ypm* genes	*ucn* genes	*sgd* genes
HxIR family transcriptional regulator	*dynU8*	*tnmR1*	*ypmR1*	*ucnR1*	*sgdU8*
AarF/ABC1/UbiB kinase family protein	*orf18*	*tnmR2*	*ypmR2*	*ucnR2*	
AarF/ABC1/UbiB kinase family protein	*dynR3*	*tnmR3*	*ypmR3*	*ucnR3*	*sgdR3*
AraC family transcriptional regulator		*tnmR4*		*ucnR4*	
AraC family transcriptional regulator	*dynR7*	*tnmR7*	*ypmR7*	*ucnR7*	*sgdR7*
Transcriptional regulator	*orf13*		*ypmR6*		
AfsR/SARP-type regulator	*dynR2*		*ypmR5*		*sgdR2*
Putative transcriptional regulator	*dynR8*				
Two-component system sensor kinase	*dynR10*				
ArsR family transcriptional regulator			*ypmR4*		

Table S4. Transcription unit analysis of *tnm*

intergenic region	the pattern of adjacent genes	length	RT-PCR selection
tnmE6-tnmR7	head to tail	35	-
tnmR7-tnmB	head to tail	205	√
tnmB-tnmR3	head to head	206	√
tnmC-tnmD	head to tail	88	√
tnmD-tnmF	head to tail	27	-
tnmF-tnmG	head to tail	153	√
tnmG-tnmH	head to tail	15	-
tnmH-tnmE10	tail to tail	73	-
tnmE5-tnmE4	head to tail	9	-
tnmE4-tnmE3	head to tail	225	√
tnmE3-tnmI	head to tail	257	√
tnmI-tnmR1	head to tail	92	√
tnmR1-tnmJ	head to head	165	√
tnmJ-tnmK1	head to tail	30	-
tnmK2-tnmL	head to tail	16	-
tnmL-tnmM1	tail to tail	70	-
tnmM2-tnmN	head to head	193	√
tnmN-tnmO	head to tail	47	-
tnmP-tnmR2	head to tail	73	√
tnmR2-tnmQ	tail to tail	52	-
tnmQ-tnmS1	head to tail	7	-
tnmS1-tnmT1	head to tail	113	√
tnmT1-tnmS2	head to tail	27	-
tnmR4-tnmT2	head to tail	88	√
tnmT2-tnmS3	head to tail	119	√

Fig. S1. The conserved domain analyses of five putative Tnm regulators

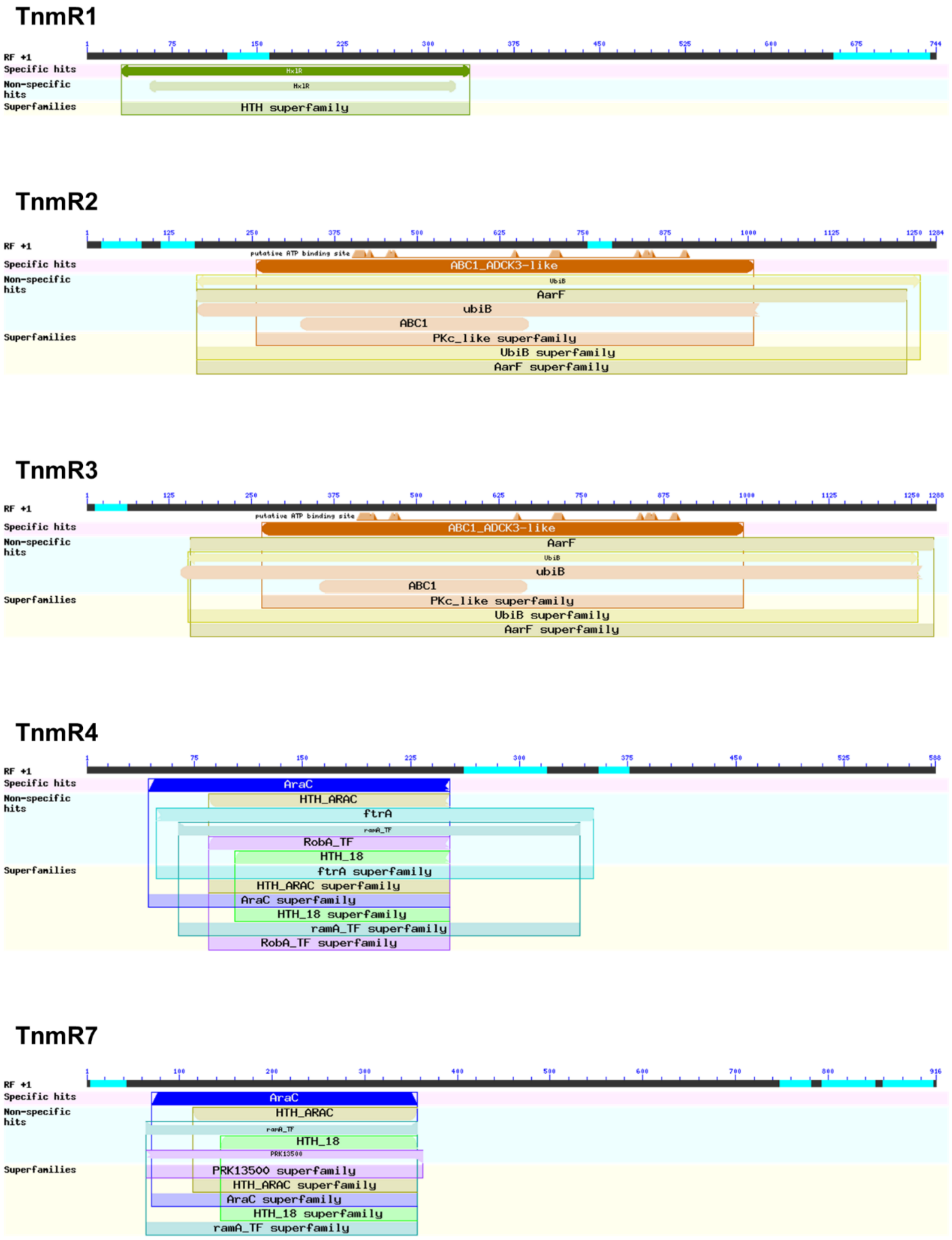

Fig. S2. The construction and verification of target regulatory gene knockout mutant derived from CB03234-S.

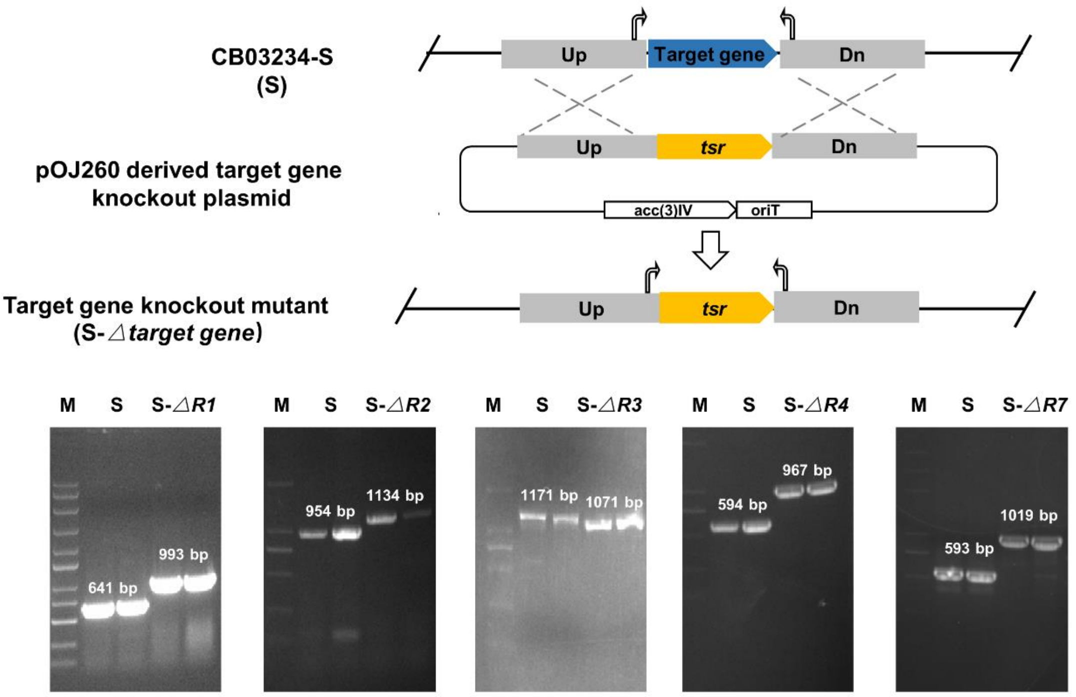

M: DNA marker;

Target gene: *tnmR1 (R1)*, *tnmR2 (R2)*, *tnmR3 (R3)*, *tnmR4 (R4)*, *tnmR7 (R7)*.

Fig. S3. The sequence similarity network (SSN) analysis of putative pathway-specific regulatory genes from five reported AFE BGCs (E-value threshold 1×10^{-20})

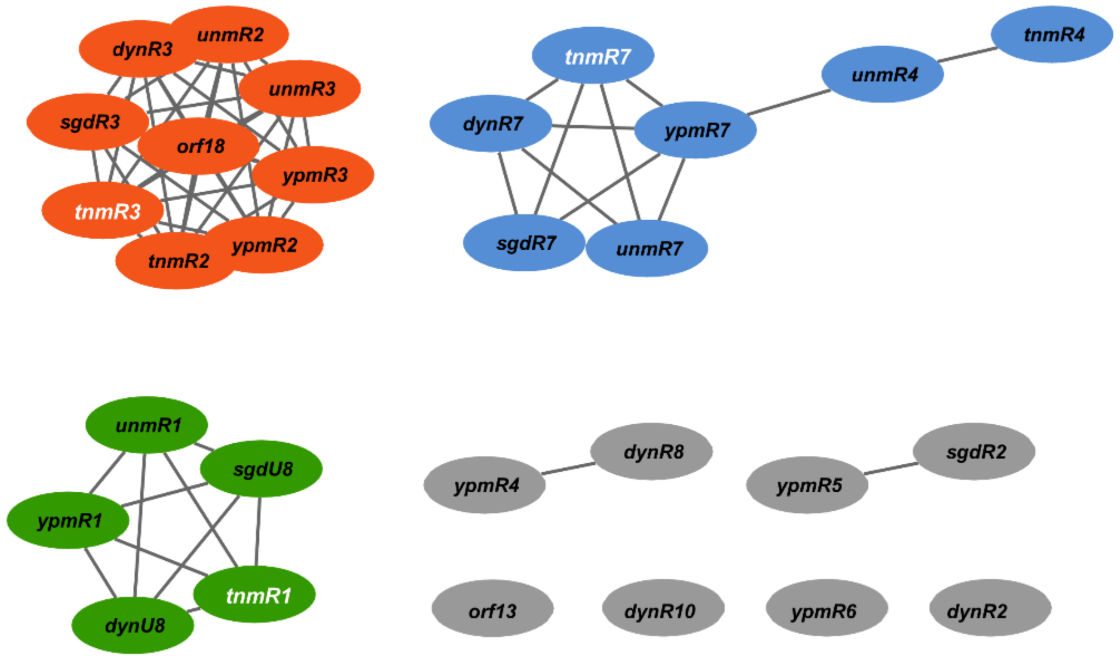

Fig. S4. Determination of transcription unit in *tnm* by RT-PCR (cDNA), using the genomic DNA (gDNA) as the reference (the co-transcription regions were marked red).

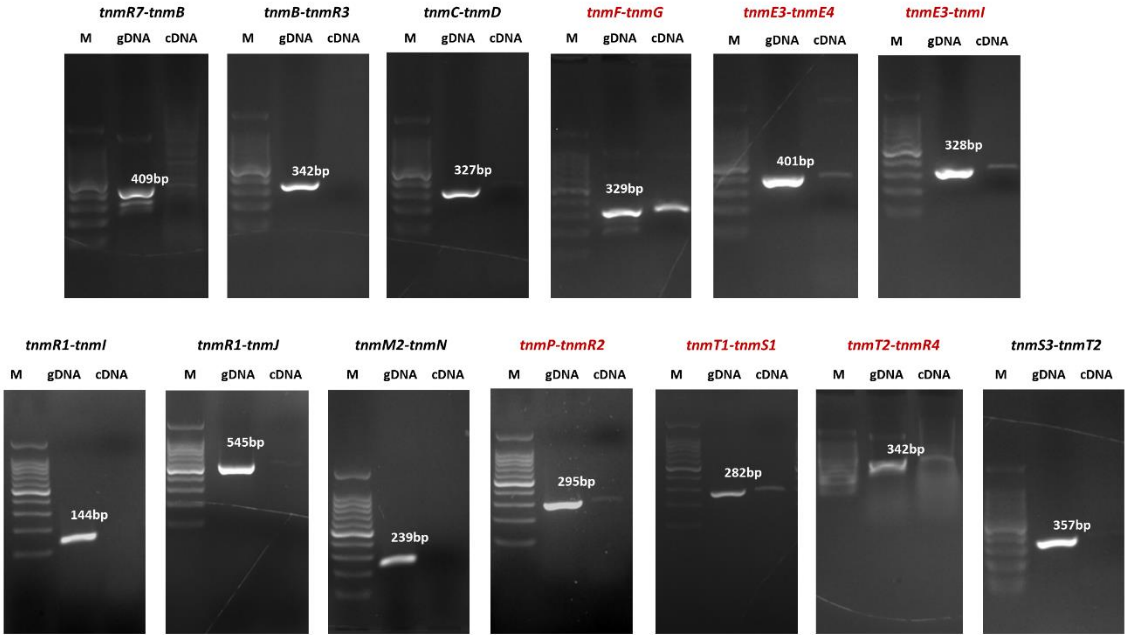

Fig. S5. The q-PCR analyses of *tnmR2* and *tnmR4* related mutants.

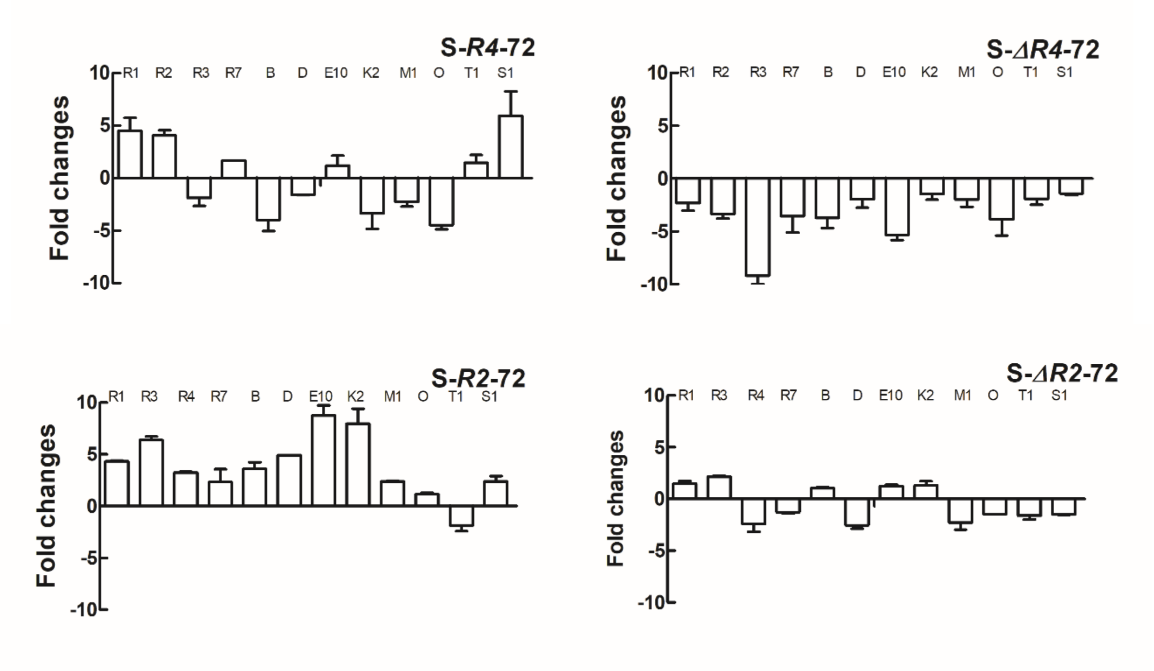

Fig. S6. The SDS-PAGE analyses of soluble TnmR1-His6, insoluble TnmR7-His6, and resolubilization of TnmR7-His6.

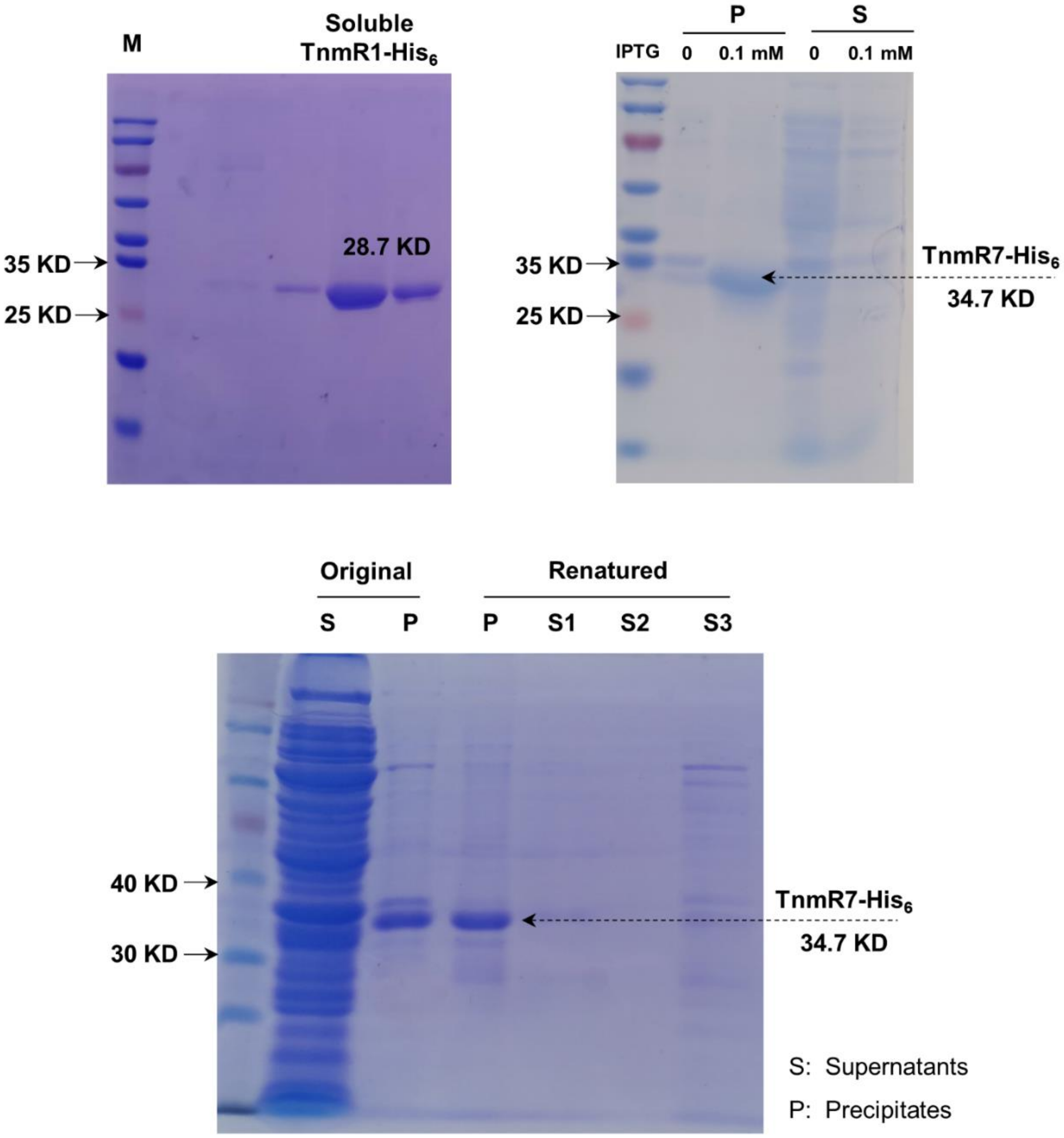

Fig. S7. A. The hydrophilicity evaluation of TnmR7 (red box indicates the hydrophobic region), and the design and SDS-PAGE analyses of the three truncated TnmR7 fragments; B. The SDS-PAGE analyses of TnmR7 with molecular chaperones or in pGEX-2T fusion system.

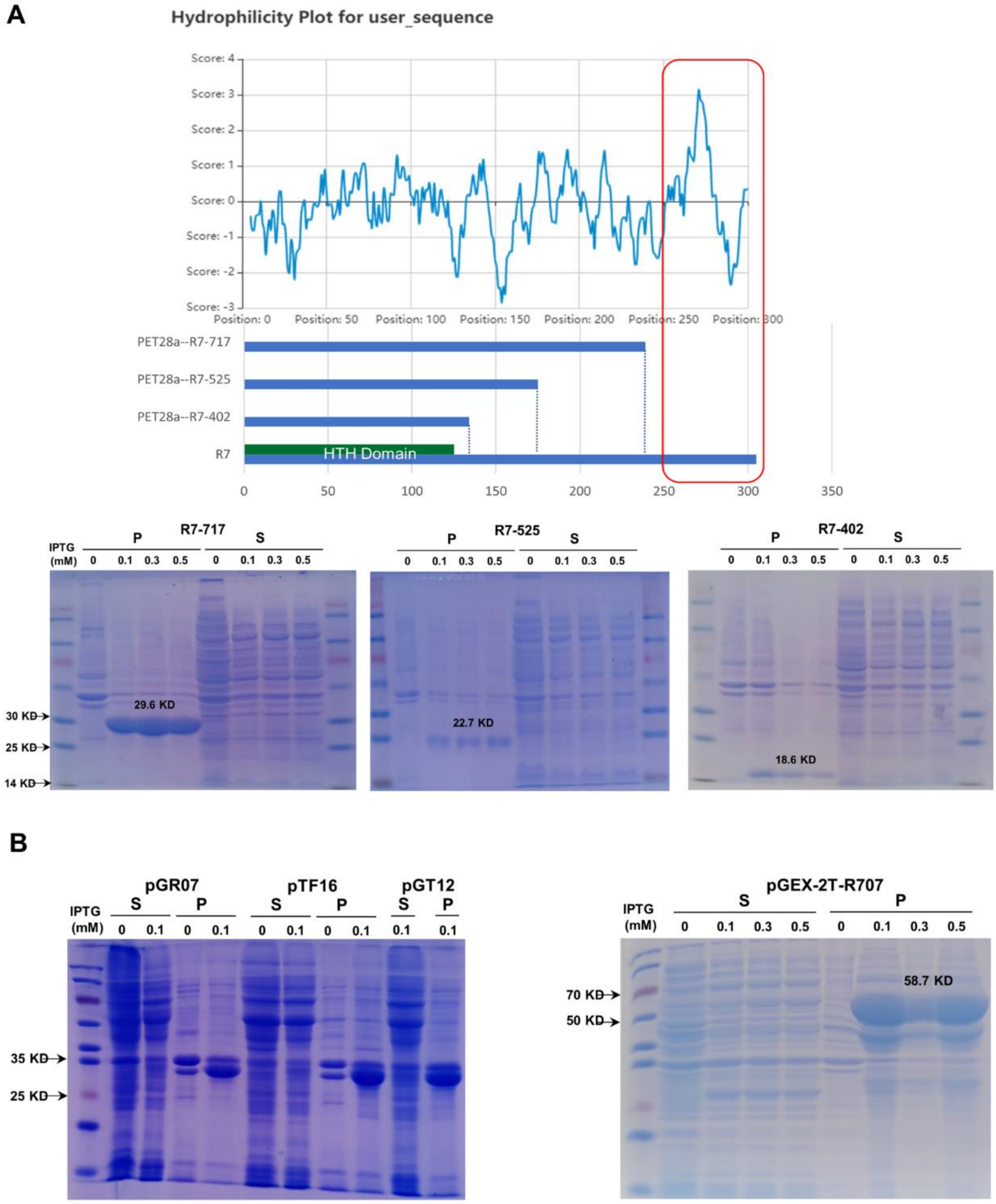

Reference:

1. Yan X, Chen JJ, Adhikari A, Yang D, Crnovcic I, Wang N, Chang CY, Rader C, Shen B: **Genome Mining of Micromonospora yangpuensis DSM 45577 as a Producer of an Anthraquinone-Fused Enediyne.** *Org Lett* 2017, **19:**6192-6195.
2. Zhuang Z, Jiang C, Zhang F, Huang R, Yi L, Huang Y, Yan X, Duan Y, Zhu X: **Streptomycin-induced ribosome engineering complemented with fermentation optimization for enhanced production of 10-membered enediynes tiancimycin-A and tiancimycin-D.** *Biotechnol Bioeng* 2019, **116:**1304-1314.
